# Counter-Narratives of Structural Oppressions, Stigma and Resistance, and Reproductive and Sexual Health Among Youth Experiencing Homelessness

**DOI:** 10.1177/10497323221110694

**Published:** 2022-06-23

**Authors:** Emilia Henriques, Catherine Schmidt, Rachael Pascoe, Kira Liss, Stephanie Begun

**Affiliations:** 1 Factor-Inwentash Faculty of Social Work, 7938 University of Toronto, Toronto, ON, Canada

**Keywords:** pregnancy, parenting, homelessness, qualitative, stigma, resilience, resistance

## Abstract

Youth experiencing homelessness (YEH) face myriad injustices regarding their reproductive and sexual health and rights. Reproductive and sexual health research with YEH often explores condom-use and sexually transmitted infections, potentially contributing to narrow conceptualizations of YEH as “unclean” or in need of disease-screening. A narrative theory perspective was applied to this study, which allowed for the emergence of alternative storylines, or counter-narratives, which attend to manifestations of power and oppression within the lives of marginalized individuals. Qualitative interviews engaged 30 young people (ages 18–21) accessing shelter services. Narrative analyses identified YEHs’ documentations of dominant narratives related to structural oppressions, stigma, and numerous dimensions of reproductive and sexual health including how they create, through their resistance, counter-narratives that include their preferred futures. YEH emphasized systemic sources of stigma and outlined their criticisms of the state. Within-group stigma emerged as a noteworthy theme, with YEH showing both empathy and ambivalence towards other YEH, along with internalization of stigmatizing narratives about pregnancy and homelessness. Approaches to service provision that further amplify youths’ voices in naming and challenging the many oppressions and stigmas they face should be prioritized. Moreover, policies should be implemented to dismantle the actual root causes of challenges faced by YEH, rather than perpetuating them through measures rooted in interlocking oppressions of discrimination, inequity, and judgment.

## Introduction

In the USA, over 3 million young people between the ages of 18 and 25 experience homelessness each year (Morton et al., 2017, 2018). The broadest federal definition of youth homelessness includes any “individual who is less than 21 years of age, for whom it is not possible to live in a safe environment with a relative, and who has no other safe alternative living arrangement” (42 U.S.C. § 5732). The majority of youth experiencing homelessness (YEH) have left unsafe home environments in which familial hostility and discord (Gaetz et al., 2016; Webb & Gazsco, 2017), physical and sexual abuse (Coates & McKenzie-Mohr, 2010; Tyler & Schmitz, 2018), negative involvement with child welfare systems (Gaetz et al., 2016; Webb & Gazsco, 2017), and disownment from families of origin based on gender identity, gender expression, and sexual orientation are familiar occurrences (Abramovich, 2012; Rosario et al., 2012).

Youth experiencing homelessness face an array of injustices pertaining to their reproductive and sexual health and rights, and multiple barriers to accessing sexual health care (Begun, 2014, 2015, 2017, Begun et al., 2019a, 2019b, 2020b, 2020a; Crawford et al., 2011; Thompson et al., 2016). In a US Midwest-based study, 70% of YEH engaging in sexual activities experienced pregnancy during their adolescence (Crawford et al., 2011). Additional research reports that 30%–60% of young women become pregnant while unstably housed (Dworsky et al., 2018; Winetrobe et al., 2013). Compared to pregnant youth with stable housing, pregnant YEH are less likely to receive prenatal and general reproductive health care (Baggett et al., 2010; Thompson, et al., 2008). As a result, they experience high rates of pre-term birth, and their babies are at greater risk of low birth weights, neurological, and/or physical complications (Chapman et al., 2007; Little et al., 2005; Oliveira & Goldberg, 2002). And acute and chronic health problems High rates of pregnancy are perhaps unsurprising given overall low rates of contraception use among YEH; research has shown that over 40% of sexually active YEH used no form of contraception in the prior year (Arangua et al., 2005), and 40%–70% of YEH (all genders) reported recent engagement in condomless sex (Barman-Adhikari et al., 2017; Tucker et al., 2012). Many YEHs’ sexual activities are influenced by pervasive conditions of community violence, sexual coercion and victimization, gender-based power imbalances, and survival sex (Haley et al., 2004; Warf et al., 2013). YEH, consequently, show higher rates of sexually transmitted infections (STIs), including HIV, compared to housed youth (Beech et al., 2003). Recent participatory research with female YEH highlights the importance youth place on being able to make their own decisions about their bodies, and the necessity of using a reproductive justice approach in sexual health interventions for YEH (Aparicio et al., 2021).

Youth homelessness is a violation of basic human rights and reflects structural failings that hamper young peoples’ access to sufficient housing, food, justice, education, health care, meaningful employment, and freedom of expression at a critical time of physical, social, cognitive, and emotional development (Canadian Observatory of Homelessness, 2017). Yet, despite the structural causes that lead to the problem of youth homelessness, YEH experience considerable stigma and judgment from broader society, and the negative consequences for their health and wellbeing have been well documented (Farrugia, 2011; Karamouzian et al., 2017; Kidd, 2007; Roschelle & Kaufman, 2004). Stigma creates and exacerbates barriers to accessing health services (Karamouzian et al., 2017) and people experiencing homelessness report frequent stigmatizing experiences with health care providers, including being disrespected, dismissed and dehumanized (Moore-Nadler et al., 2020). Moreover, studies of YEH indicate a strong connection between perceived stigma and negative mental health outcomes, including low self-esteem, loneliness, feelings of hopelessness and helplessness, and suicidal ideation (Kidd, 2006, 2007). Kidd (2007) found that guilt and self-blame due to homeless status had a particularly strong relationship with negative mental health outcomes, concluding that the degree to which YEH internalize stigma may impact how detrimental it is to their mental health.

In contrast, narratives of strengths, hope, and resistance to stigma among YEH, especially with regard to their reproductive health, have been less documented. The purpose of this qualitative study was to better understand YEHs’ experiences of and responses to stigma within their lives, with a focus on how such experiences influence their attitudes towards pregnancy and reproductive health. Stigma is defined as the “discrediting mark” or attribute in the form of negative attitudes (i.e., prejudice), beliefs (i.e., stereotypes), behaviors (i.e., discrimination) assigned to an individual or group, resulting in reduced perception of status and worth (Chambers et al., 2015; Firmin et al., 2017). Goffman (1963) defines stigma as a disreputable characteristic that spoils one’s identity. In this study, we consider both *individual stigma*, focused on individual perceptions and how these shape micro-level interactions of discrimination, as well as *structural stigma,* focused on macro-level forces, or the “societal-level conditions, cultural norms and institutional policies that constrain the opportunities, resources and wellbeing of the stigmatized” (Hatzenbuehler & Link, 2014). Based on interviews with 30 YEH living in a shelter in an American city, this study uses a narrative theory framework to examine the dominant narratives which hold power in stigmatizing YEH, and to highlight counter-narratives of hope, resilience and resistance to systemic oppression.

### Youth Experiencing Homelessness, Intersectionality, and Stigma

Youth experiencing homelessness are a heterogeneous group with multiple intersecting identities, and an intersectional approach is therefore key in considering the impacts of both individual and structural stigma for individual youth. The term intersectionality refers to the various ways that multiple identities interact to shape dimensions of experience, such as how the experiences of women of color in the context of violence against women, are informed by racism and sexism, and are generally underrepresented within antiracist and feminist discourse (Crenshaw, 1990). In the case of homelessness research, understanding intersecting marginalized groups, such as racial minorities, women, LGBTQ+ individuals, and youth, is important for understanding the causes of homelessness for these populations and for challenging the homogenization of “homeless” as a group (Lurie, et al., 2015).

In addition to the poverty and class-based oppression experienced by YEH, individual experiences of youth homelessness may be shaped by racism, sexism, heterosexism, homophobia and transphobia, and able-ism, as well as other forms of structural oppression. Research shows that lesbian, gay, bisexual, transgender, queer/questioning, and other individuals identifying as sexual and gender minorities (LGBTQ+) are overrepresented in YEH populations in the USA (Durso & Gates, 2012), as are Black youth (Gattis & Larson, 2017). Racialized and LGBTQ+ YEH experience unique challenges that their white, cis-gendered and straight peers do not experience (Gattis & Larson, 2017). For example, Black LGBTQ YEH are more likely to experience both overt violence and other forms of discrimination, including police and community harassment and criminalization (Reck, 2009). In their study of LGBTQ+ YEH engaged in survival sex, Alessi et al. (2021) found that participants often felt unsafe within shelters and social service systems due to the homophobia and transphobia they experienced. Further troubling, transgender participants described facing additional discrimination when attempting to find housing and employment, and participants who were both transgender and racialized reported instances where they felt targeted by police as a result of their intersecting identities (Alessi et al., 2021).

### Youth Experiencing Homelessness Responses and Resistance to Stigma

Responses to experiences of stigma are varied. YEH may internalize stigma, leading to feelings of guilt and shame that can negatively impact mental health (Kidd, 2007). Stigma may also lead some to respond through forms of within-group stigma, including processes of “distancing,” which Lott (2002) describes as the exclusion, separation, devaluing, and discounting of an individual or group, and “othering,” defined as the classifying of a group as inferior, whilst simultaneously providing restricted advantages, such as resources, to the dominant group. Alternatively, YEH may show resistance to stigma in a myriad of ways. Firmin and colleagues (2017) describe resistance to stigma as the process of being unaffected by stigmatizing attitudes, actively challenging stigma, and holding a positive identity grounded in strengths despite stigmatizing conditions. Resistance to stigma occurs on a personal level, such as catching and challenging stigma within one’s own thought processes, empowering oneself, and developing a meaningful identity and purpose; stigma resistance also takes place with peers, such as sharing lived experiences and offering informal peer support (Firmin et al., 2017). On a public level, structural stigma may be challenged through confronting and questioning stigmatizing policies, educating others, and disclosing one’s own lived experience through advocacy (Firmin et al., 2017).

Recent research with LGBTQ+ youth experiencing homelessness illustrates the range of ways in which youth show resilience and resist stigma, despite the many challenges they encounter (Alessi et al., 2021; Schmitz & Tyler 2019). Youth resist stigma by living as their authentic selves despite pressures to conform to heteronormative norms, by creating strong bonds of support with other YEH, and by standing up to oppressive systems when their rights are disrespected (Alessi et al., 2021; Schmitz & Tyler, 2019). Our study builds on and contributes to emerging literature emphasizing youth resilience and resistance, using a narrative theory approach.

### Narrative Theory

Narrative theory was applied to this study because of its applications for conceptualizing lived and preferred stories of disenfranchised communities when navigating stigma. While narrative theory posits that no sole narrative is a single truth considering the multiplicity of interpretations, certain narratives wield more or less power based on the extent to which they circulate within society, allowing them to become either dominant- or counter-narratives (McKenzie-Mohr & Lafrance, 2017; McTighe, 2018; Williams & Baumgartner, 2014).

Many dominant storylines regarding homelessness are laced with stigmatizing descriptions whereby homelessness is positioned as a choice caused by intrinsic failings (Williams & Baumgartner, 2014). Dominant narratives tend to be problem-saturated with conceptualizations of those experiencing homelessness as mentally ill, addicted to substances, lazy, idle dangerous, delinquent, deficient in skills and abilities, and untrustworthy (Toolis & Hammack, 2015). The ongoing devaluation of unhoused people is subtly yet systematically incorporated into the psyches of society at-large through the circulation of such dominant narratives (Toolis & Hammack, 2015). Such dominant narratives obscure the role that broader social forces of racism, classism, and heterosexism play in limiting socioeconomic upward mobility, while further minimizing the inherent strengths of YEH (Toolis & Hammack, 2015).

Similarly, dominant narratives tend to depict those who are pregnant or parenting while homeless as being irresponsible and more deserving of scrutiny and surveillance with regard to their approaches to parenting; these beliefs lead to increased child welfare involvement, parent-child separation, and feelings of degradation (Fraenkel et al., 2009; Holtrop et al., 2015). In contrast to these problem-saturated narratives, pregnancy and parenting have been illustrated by some YEH as motivating factors for establishing positive life changes, such as finishing school, reducing substance use, or obtaining housing and employment (Begun, 2015, 2017; Begun et al., 2019a; Hathazi et al., 2009). Pregnancy is viewed by some YEH as an opportunity to create emotional connections as a means of reimagining a future beyond the fractured bonds that many YEH experienced in their families of origin (Begun, 2015, 2017, Begun et al., 2019a, 2019b; Thompson et al., 2008; Tucker et al., 2012; Winetrobe et al., 2013).

Given the role of academic scholarship and knowledge production in shaping dominant narratives, research about marginalized groups can sometimes, even if inadvertently, contribute to further stigmatizing and marginalization. For example, research on sexual and reproductive health among YEH generally concerns condom use and sexually transmitted diseases (Caccamo et al., 2017), potentially contributing to narrow conceptualizations of YEH as “dirty,” “unclean,” and in need of screening for diseases. In contrast, using a narrative theory perspective allows for alternative storylines, known as counter-narratives, which attend to manifestations of power and oppression within the lives of marginalized individuals. Research focused on narrow conceptions of resilience, which highlight individual strength and successful assimilation into dominant norms of society, can leave oppressive social structures unexamined and contribute to upholding dominant relations of power within society (Robinson & Schmitz, 2021). As an alternative, a narrative theory approach considers problems as residing within the broader social, economic, political, and historical contexts, and counter-narratives act as a platform for stigmatized individuals to resist harmful storying of their lives, while seeking liberation from disenfranchisement and insubordination (McKenzie-Mohr & Lafrance, 2017; Williams & Baumgartner, 2014). Counter-narratives in research with YEH have been captured through instances wherein YEH have questioned the status quo, identified structural barriers and inequities, externalized problems to residing within the broader oppressive social and political forces, and re-claimed their narratives through crafting and restoring a meaningful identity and sense of purpose, pride, hope, and empowerment (Toolis & Hammack, 2015).

Youth experiencing homelessness, particularly those with additional intersectional marginalizations, are acutely aware of dominant narratives and the role they play in perpetuating disempowerment, social isolation, anti-homelessness policies, criminalization, and exposure to dangerous circumstances (Kidd, 2007; Toolis & Hammack, 2015). As such, this paper uses a narrative theory perspective to explore how YEH ascribe meaning to their experiences of and resistance to stigma in their daily lives and more narrowly with regard to their reproductive health.

## Materials and Methods

This study was part of a larger one that examined the perspectives and experiences of YEH regarding pregnancy and parenting, and reproductive and sexual health. The research questions guiding the current analysis were as follows: (1) How do YEH experience and make meaning of individual and structural stigma in their daily lives? (2) How do experiences of stigma influence their perspectives regarding pregnancy, family planning and sexual and reproductive health? (3) How do youth respond to, internalize and/or resist such stigma?

### Recruitment and Study Participants

Criterion sampling, an approach deemed appropriate for studies in which all participants experience the phenomenon under study (Saldaña, 2013), was used to identify youth and young adults residing at an overnight youth-serving shelter in Denver, Colorado. Criterion sampling involves identifying participants based on a set of criteria, which this study defined as young people whose experiences matched broader federal and agency-based definitions of youth homelessness. Most youth served by this host organization were under age 21 and per the agency’s preference of not including minors in this study, which the agency served very infrequently and typically through customized services and supports, only young people ages 18 and older were recruited to participate in the current study. Youth of all genders were invited to participate to gather the most diverse and intersectional perspectives and experiences among youth available.

Youth were approached in the shelter milieu by the study’s Principal Investigator (PI), a social work researcher (white, cisgender, able-bodied woman, a parent, and with no lived experience of homelessness), who at the time was frequently present in the shelter for data collection across several concurrent research studies. As the PI spent considerable time at breakfast, lunch, and “hang-out” times with young people at the shelter, most youth present at the time of the current study were familiar with the researcher and had interacted with her during such gatherings. The study purpose was described to youth as being a project that sought to examine the reproductive and sexual health attitudes and needs for supports specifically among YEH, and with acknowledgment that these concepts can mean a countless array of things depending on who is asked and that this study sought to collect and honor a diverse range of insights that were rooted in contributors’ unique identities and lived experiences. The researcher also named upfront that she had extensive work experience and interest specifically in areas of reproductive rights and reproductive justice, LBGTQ+ health and rights, and youth-led organizing, and that her education goals and research were similarly rooted in these areas. To participate, youth were required to provide written informed consent indicating their willingness to engage in the research interview. All study details were approved by the PI’s university-based Institutional Review Board. Permission to audio-record interviews was obtained from all participants. Participants were then engaged in individual interviews, lasting 45–60 min, and facilitated in a private office in the shelter by the study PI. Respondents received a $25 gift card to a general retailer or local food vendor.

### Data Collection: Qualitative Interviews

A semi-structured interview guide was used to explore participants’ attitudes and experiences regarding pregnancy and parenting, family planning, their needs for information and social supports, and other topics of reproductive and sexual health (e.g., abortion, contraception, sexually transmitted infections (STIs), reproductive and sexual health knowledge and service utilization experiences). An introductory, hypothetical question was posed at the beginning of each interview, and was followed by a series of potential prompts that were utilized as relevant and dependent upon respondents’ answer trajectories. At the beginning of each interview, a question was asked: “To get started, I’m going to ask you some hypothetical questions. If you were to wake up tomorrow and learn that you are pregnant or got someone pregnant, how do you think you’d react?” From there, participants’ perspectives dictated interview flow, and the semi-structured guide offered a general framework for asking participants overall consistent questions. Although the precise order of each interview was unique to the youth respondent because of their answer trajectories and in wanting the conversation to flow freely and comfortably, the researcher was nonetheless intentional about making sure that topics of abortion, contraception, STIs, parenting, social support, health care utilization, and social norms regarding reproductive and sexual health were approached in each interview in at least some capacity. At the close of each interview, participants were asked to complete a brief, voluntary, pen-and-pencil survey to collect sociodemographic information to assist in describing the sample. Participants answered several questions, such as pregnancy history, foster care history, homelessness duration, and how many cities they had lived in since leaving their home of origin (see Table 1). Sampling concluded after thematic saturation was reached, as participants’ responses appeared to be increasingly consistent as the sample size surpassed approximately 25 interviews. Data collection concluded after 30 interviews, giving all eligible youth residing at the shelter during that time an opportunity to participate. Pseudonyms were assigned to participants using ambiguous multicultural names from an online random name generator.

**Table 1.** Sample Characteristics of Homeless Youth in City Blinded (*N* = 30).

Characteristic	*n*	(%)
Gender		
Man/Male	10	(33.3)
Transgender or gender-non-conforming (e.g., gender queer, trans man, trans woman)	4	(13.3)
Woman/Female	16	(53.3)
Race/ethnicity		
American Indian or Alaska Native	1	(3.3)
Black	6	(20.0)
Latino/a	3	(10.0)
Multi-racial	6	(20.0)
Native Hawaiian or Pacific Islander	1	(3.3)
White	13	(43.3)
Sexual orientation		
Bisexual	2	(6.7)
Gay	2	(6.7)
Lesbian	2	(6.7)
Pansexual	4	(13.3)
Queer	1	(3.3)
Questioning	1	(3.3)
Straight	18	(60.0)
Foster care history (yes)	9	(30.0)
Currently pregnant (yes)	4	(13.3)
Pregnancy attitudes		
Anti-pregnancy	12	(40.0)
Pro-pregnancy or pregnancy-ambivalent	18	(60.0)
	M	SD
Age (years)	19.1	0.8
Time homeless (months)	8.9	9.0
Transience (number of cities lived in since leaving home)	2.5	2.0

### Data Analyses

Qualitative data were initially analyzed utilizing a phenomenological research epistemology with thematic analysis (Fraenkel et al., 2009), which is considered suitable for the investigation of topics that are not well understood to seek more profound meaning about an experience or circumstance that a group of individuals shares (Padgett, 2011). The broader “phenomenon” researched through this project was youths’ experiences of homelessness, specifically as young people contending with reproductive and sexual health information-seeking and decision-making amidst accessing temporary shelter-based services. In more focused, subsequent analyses, a narrative theory approach was used to examine youths’ accounts of stigma and resistance. Derived from post-modernism, which posits that one’s understanding of the world is contingent upon time and culturally-bound meanings, and social constructivism, which suggests that the environment shapes individuals just as individuals shape the environment, narrative theory assumes that the stories or narratives that one tells about themselves, and that are told about them, are central to how an individual understands, maintains, and gives meaning to their existence (McKenzie-Mohr & Lafrance, 2017; McTighe, 2018; Williams & Baumgartner, 2014).

Qualitative interviews were transcribed and uploaded to Dedoose, the secure, cloud-based, qualitative analysis software platform. After initial qualitative thematic analysis by the PI, additional analyses were conducted by a team of four research assistants, who worked independently in order to reduce bias and increase rigor in analyses. The research assistant team was comprised of social work graduate students (two MSW and two PhD students), who identified as predominantly middle class, white, cisgender female, and who had a range of one to 10 years of clinical social work experience. The team was comprised of individuals with other intersecting identities and lived experiences, including parents and those who had experienced pregnancy, Jewish ethnicity, and an array of sexual orientations represented. The team had limited personal histories of experiencing homelessness, though had worked in various frontline service environments with individuals experiencing homelessness and other marginalizations.

From a secondary examination of the data by research assistants, an additional area of investigation was identified, distinct from the original scope and goal of the interviews: that of stigma and resilience demonstrated in relation to youths’ experiences of homelessness and reproductive health. Additional topics, themes, and their examination are detailed elsewhere (Begun, 2017; Begun et al., 2019c, 2019a, 2019b, 2020b, 2020a). Following initial independent coding, coders convened to compare codes generated and applied to transcripts, discussed the appropriateness of the coding structure developed, resolved discrepancies, and established consensus in analyses. A thematic analysis approach was used in analysis, with a first stage entailing line-by-line open coding followed by a re-evaluation of preliminary codes and emerging themes and concluded with a final round of “focused coding,” whereby the most salient themes were identified and organized (Saldaña, 2013).

## Results

### Sociodemographic Characteristics

As shown on Table 1, 53.3% (*n* = 16) of participants in the sample identified as women, 33.3% (*n* = 10) as men, and 13.3% (*n* = 4) as transgender, gender-non-conforming, or gender-fluid. The sample was also diverse regarding racial identity and sexual orientation; 56.7% (*n* = 17) of participants identified as youth of color, and 40% (*n* = 12) identified with sexual orientations other than straight/heterosexual. On average, participants were 19.1 (*SD* = 0.8) years old and had experienced homelessness for an average of 8.9 (*SD* = 9.0) months. Participants had lived in an average of 2.5 (*SD* = 2.0) cities since leaving their home or place of origin. Nine youth (30%) had been in foster care, and four youth (13.3%) indicated that they were currently pregnant, information that they voluntarily disclosed, unprompted, within their respective interviews.

### Theme 1: Stigma as Described by Youth Experiencing Homelessness

#### Sources of stigma

Experiences of stigma and discrimination were common among YEH interviewed. Sources of stigma, described as judgment or negative perceptions about homelessness, existed both within and outside of YEH communities. Stigmatizing perceptions described by participants mirrored those seen in extant literature, such as perceptions of laziness, lack of motivation to care for themselves, and inability to plan for the future. Experiences of stigma were noted in instances when participants felt excluded or neglected, including rejection by family members and friends, and negative interactions with health professionals and other service providers.

Several participants described experiences of stigma and judgment from health-care workers, particularly doctors, nurses, and staff at reproductive health centers. YEH reported having their statements to medical staff questioned based on stigmatizing assumptions about their sexual practices: “Yeah, [doctor will be thinking] here’s an 18-year-old girl staying at a shelter and has like three potential baby daddies and is a mess. Great” (Arden). Having access to services that made efforts to reduce stigmatizing language were heralded as particularly helpful if they “…told people in a way that didn’t make them feel like it was a lecture or that we’re all a bunch of dumb homeless kids, that would be a start” (Kiran).

Unsupportive family members were often the source of participants’ feelings of stigma, especially regarding LGBTQ+ status: “I no longer talk to [my parents]. They kicked me out when I came out, so we’ve been on thin ice for years” (Reese). Some participants also reported the loss of friendships or community resulting from homelessness stigma:

I don’t think those relationships hold up very long for most of us. Most people who aren’t homeless judge people who are homeless, even if their situation for becoming homeless is really honest and sad. There’s just a lot of discrimination about homeless people, so those friends usually don’t really stay friends (Jayme).

Stigma towards YEH was also identified within society more broadly. Government policies, the words and inaction of individual politicians, and a lack of access to services were described as sources of stigma, which were particularly salient considering recent governmental decisions at the time of interviewing. Participants also reported feelings of stigma as a more amorphous sense that society is generally hostile towards YEH. Participants discussed feelings of stigma in terms of specific reactions from strangers, or from a general sense of stress and judgment they experienced in the community: “The reason I’m homeless has nothing to be with being an irresponsible, freeloading type of person like most people probably think” (Lavern).

#### Stigmatizing dominant narratives: feeling unseen and unclean

An overarching theme described by many of the participants was that of being “unseen” and “unclean.” Youth homelessness was an issue that many choose not to see or address, and that when seen, YEH were considered dirty and diseased. Participants often described themselves and other YEH as “invisible” and expressed feelings of being forgotten or unacknowledged: “I think our challenges are really forgotten, or not even known, by most people…” (Mattie). Some participants expressed that being listened to while being interviewed felt like a novel experience because they are so used to be ignored: “I realized when I was talking that I’m so forgotten now, especially as a homeless person, that people don’t even really ask me what I think or feel about some of these important things” (Swarna).

Feelings of being perceived as unclean, both by health-care providers and by the general population, were often described by participants in reference to their sexual and reproductive health. For instance, one participant described:

Getting birth control is just sort of weird because then the doctor judges, like why are you homeless and having sex? You could get pregnant, don’t you think that’s a little irresponsible at this time?.. It’s just a painful time, going to the doctor. I feel like I’m like this leper of society. I feel like they look at people like me and just think ‘well, she’s garbage' (Chidi).

Participants also described that their STI status was often assumed by their health-care provider. For instance, one participant described how an ER doctor’s assumption that her recurring bacterial vaginosis was an STI:

And the ER doctor was such a jerk. He said, ‘well, you probably just have another STD.’ And I was like ‘Excuse me, I don’t have any STDs, and I know what this is. It has been diagnosed, and treated, but it can still come back.' And I felt so disrespected. And judged (Jordan).

Another participant described similar assumptions on the part of potential sexual partners: “the other person assumes the homeless person has diseases.” (Mattie). Stigma within health care settings was profound across many interviews, though was particularly compounded for participants with intersecting marginalized identities. For example, one participant described the difficulty in finding a doctor “who gets how to work with LGBT people. I mean, so many doctors don’t. They’re all weird like we have the plague or something.” (Reese).

#### Youth experiencing homelessness pregnancy-specific dominant narratives

Stigma related specifically to pregnancy was described throughout the interviews, both by participants who had experienced or observed instances of judgment and exclusion due to pregnancy, as well as by participants who described their own judgment and negative stereotypes of pregnant YEH; the complexities of this within-group stigma are further explored in the following section. Stigmatizing experiences related to pregnancy appeared to be exacerbated by homelessness and age for those interviewed. Participants who had experienced pregnancy recounted being judged negatively by health-care providers, family members, peers, and society at-large. Participants reported that even within the YEH community, pregnancy often signaled a transition in friendships as, “whoever’s not your real friend will immediately start talking shit. During your pregnancy you will lose every single fake person. It will start the moment you announce, and it will end the day that the baby is born” (Jae).

For many YEH, pregnancy was viewed as an indicator of laziness and irresponsibility, connecting to broader dominant negative narratives about this group. In discussing YEH who are pregnant, one participant shared this dominant narrative: “They’re just too lazy to get a job and just do not try to get help the right way. Pregnancy is a short-cut. Like 'I can get insurance if I’m pregnant” (Jayme). These labels of laziness and irresponsibility were also identified as being felt by youth who were pregnant at the time of interview: “I also feel a little judged, like people are throwing shade at me behind my back about being pregnant, like I’m some irresponsible hoe or something” (Hira). A connected dominant narrative concerning YEH pregnancy was that young women become pregnant to obtain access to more public services and supports, and to take advantage of the system. Within the shelter system, participants stated that pregnant YEH would often be given access to additional services: “Some think they’ll get more stuff for free. And I guess you do get better access to things [*sic*] like food and even housing…” (Cruz).

Pregnancy-related stigma described by participants was clearly gendered, falling almost entirely on female YEH, and was connected to the stigma female YEH experience for being sexually active or promiscuous. This stigma was observed as impacting young women trying to access STI testing (i.e., “because they’re getting tested then there’s a reason they need to be tested, like for being a hoe or whatever” (Chantrea) as well as women who became pregnant or had an abortion (i.e., “everyone will be like, ‘Oh, she’s a hoe. She got pregnant. She’s had abortions… It’s like you’re damaged or something if people learn you’ve had an abortion” (Tendai). Some participants observed that young, marginalized women would be judged no matter what choices they make if they become pregnant:

So it’s all on the girl. A lot of pressure, and she ends up taking all the blame, like blame for getting pregnant, like for having an abortion, like giving it up for adoption, and even for like raising the child with her life situation and struggles (Jordan).

Stigma related to sexual and gender minority identities also intersected with homelessness and pregnancy. For example, one lesbian-identified participant observed that even when she is able to escape the stigma of homelessness and poverty, she will still be seen as unfit to be a mother because of her sexual orientation: “And I think even when I’m no longer homeless, like having a job and on my way with my education, it’s still like being a pregnant lesbian is not really the right kind of pregnancy to most” (Jun). Another participant discussed how difficult pregnancy would be, as it would discount their struggles to live as their true identity, instead feeling as though they are seen only as a cisman, instead of a trans woman: “So it would be so fucked up to find out that I got someone pregnant. Because it would ruin the true image of myself that I have been trying to display for so long” (Arya).

### Theme 2: Responses to Stigma

Throughout the interviews, participants articulated or demonstrated different responses to the many forms of stigma described in the previous section. Our analysis identified numerous ways in which participants resisted stigma through their efforts to enact and tell different stories about their lives that counter dominant stigmatizing narratives.

#### Striving to tell a different story by working towards a preferred future

Participants’ hopes for their futures featured dominantly in most interviews, representing a pathway out of the daily challenges they face while experiencing homelessness. The importance of being able to envision a preferred future was described explicitly by some participants. For example, one participant described the negative mental health impacts she perceived for YEH who are unable to envision a future beyond the daily struggle of survival:

I think they’re not seeing as much in their future… It’s more just a daily exercise, trying to make it through. And there’s lots of depression, lots of drugs, drinking, things like that. A lot of people can’t really see themselves, like past themselves and the current struggles they’re having (Sam).

#### Future goals related to education and career

Many participants described the steps they have taken towards educational or career and other goals related to their future hopes. Most participants were involved in multiple activities and described how they were working towards their preferred futures for themselves: “Most of us have so much going on. So many groups to go to, always riding the bus around to our appointments, always going to GED classes or job training or case management meetings and whatnot” (Kit). Many described their aspirations primarily in relation to their educational and career goals, and for some, these were strongly connected to their attitudes towards pregnancy. For several participants, the desire to focus on their educational and career goals was articulated as a primary reason for wanting to avoid pregnancy, while others described seeing pregnancy as compatible with their other life goals.

For the first group, avoiding pregnancy was seen as an essential part of their hopes for the future and moving forward with their career and educational goals. Many participants discussed their career and/or educational aspirations in the context of the pressures that they feel to avoid negative stereotypes associated with youth homelessness or in relationship to their racial identity**.** Pregnancy was seen as a threat to their future goals that would lead them to embody the negative stereotypes they seek to avoid. They articulated their motivation to succeed, and to avoid pregnancy, as a response to this dominant narrative, and a way of telling a different story. For example, one participant who was working and studying full-time, explained why she would want an abortion if she were to become pregnant:

I just want to keep working toward my goals. I think there’s a lot of pressure, that I feel from society, to show that I am a strong, smart woman. And I’m First Nations. So that’s a big deal to me, that I’m not seen as something bad. I don’t want to be a stereotype or this person for White people to talk about negatively. I want to be Dr. [name blinded – Indigenous name/context]. Not another statistic of someone on a reservation. So if abortion is what needs to happen for me to be Dr. [name blinded] then that is the choice I would make (Sam).

Similar feelings were expressed by a participant who described her desire to avoid racialized stigma she saw as associated with young Black and Latina girls who become pregnant:

Well, so I know everyone is all like “Black girls, Latina girls, they always become pregnant.” And I don’t want to feed that stereotype by becoming pregnant really young either. And so my mom was a teen mom, and I really respect her and all but…I learned so much from her, she’s really an amazing person. But all the poverty, all of the hardship. It, um, coulda been so different, for both of us. We’ve had a lot of hard times. … I guess I just want to experience the world you know? See places, go places, learn things, meet different kinds of people, and not just be another girl. Mixed race girl, you know, getting pregnant (Jordan).

In contrast, other participants described the possibility of a pregnancy and a child as compatible with their future goals, and therefore viewed it as potentially positive. These participants described their optimism about a hypothetical pregnancy in the context of being partnered in strong relationships and their sense that having a baby was compatible with their work and educational goals. They did not describe feeling stigma attached to pregnancy, but rather a sense of pride that they could make a pregnancy work at this stage of their life, and a sense of hope for their futures. For example, one participant described feeling that having a baby could be a positive thing, though she and her girlfriend are not currently planning to do so, because of how hopeful she feels about her relationship and her career goals

And my job is pretty good… I think I’m nearly to a place that I have enough saved to get an apartment. I think I can become a manager at my job in the future, and I’m doing some freelance graphic design work. I have a few clients and I think as that takes off a bit more, I will actually be doing really well financially (Cruz)*.*

#### Family-related goals: Parenthood as an escape from stigma

Several participants discussed how becoming a parent may be perceived as an avenue to escape the stigma experienced by YEH: taking on a new role as parent provided a different, positive identity in contrast to the stereotype of “lazy homeless youth” and this could provide a new sense of pride and self-worth, new purpose for life, and a way of becoming seen by the general public as a legitimate person worthy of respect:

Having a child would be like a lot of pride, and something other people would see you, see good in you, for doing. …. It takes a lot of work to be a parent, and I think there’s a lot of honor and pride in that….People start to show them more respect, like “you’re going to be a parent, that’s important” rather than seeing them as some dumb, homeless kid (Jadyn).

Some described how becoming a good parent could be a way of showing “society” that their perception of YEH is wrong. Participants expressed that pregnancy could garner them respect in the eyes of others, given that it is a large responsibility that they are choosing to take on: “Sort of middle finger to the world, too, like ‘I can be a great parent, even if you think I’m like homeless trash” (Lavern). Another participant described how parenting would be a remarkable accomplishment given the many doubts and barriers to navigating systems and supports exist for YEH:

Like having a baby, that’s a big accomplishment. And raising it, and figuring out all of those things that need to be figured out in order to do that, that’s really impressive. And I think society thinks we can’t pull that off, but I think getting pregnant can be a way to break that stereotype, if that makes sense. (Jun).

Pregnancy was described as a way to actively reject the feelings of being unseen, as becoming pregnant or having a baby could increase the visibility of YEH in the eyes of carers and workers in their lives: “I think homeless people are often feeling invisible… so when you say you’re pregnant, you’re now a future mother, and not as much a random homeless person who people are afraid of and who seem to hate so much” (Moana).

None of the participants who were parents were living with their children at the time of their interview, but they did express their hope to be reunified with their children in the future and the importance of having their children in their lives. For example, one of the mothers described her children as motivating her recovery*:*

… I have been doing a lot of groups, getting my life in order. Working on my recovery, and getting back on my feet again. So the boys are with my mom, and I’m here at the shelter, but the shelter has given me a lot of good things. Good groups, case manager. I feel hopeful and think I can be back into a place with my kids in about a year if I keep working (Arden).

Accordingly, while some YEH described their own belief that having a child would enable them to break stereotypes or challenge stigma, others identified this line of thinking in other YEH but expressed skepticism about it, observing that YEH who become pregnant generally face new and greater challenges, and in some cases, additional forms of stigma.

#### Systemic counter-narratives—telling a different story About the sources of problems

Several participants described how systemic issues shape their own lives, or those of other YEH, and the options available to them. Shining a light on how power operates at systemic levels, these participants actively resisted dominant oppressive narratives that cast YEH as individually responsible for their poverty and life situations. In many of the interviews, participants named forms of systemic oppression they experience within their own lives, including sexism, racism, homophobia, transphobia, and classism. In some cases, participants articulated a clear link between systemic oppression and the ways in which it has shaped their own life circumstances and vision of the future. For example, a Black cis-male youth described his fears about raising his hypothetical child due to the dominance of anti-Black racism:

It’s hard to be a Black man in our society, you know, and I’m guessing my kid would look Black too…I worry every day that I’m going to be shot by a police officer. Or that someone’s going to call me some racist name. At least out in some nature area, no one is going to kill my son or call him a name (Chantrea).

Other participants asserted explicitly that it is structural issues that have led to their current situation of homelessness. For example, one participant described a sense of pride in the career and other life goals that he and his girlfriend were working towards:

I think the only things we have going against us are poverty. And racism. Because otherwise we would have what we need to not be homeless and not having to work so extra hard just to have a good life (Jayme).

#### Critiques of the state: Lack of support for low-income people and barriers to health care

Some participants discussed the state’s responsibility for injustices they perceive around them, criticizing the government’s failure to support YEH and other people living in poverty. A few participants articulated their sense that the government does not care about people living in poverty and is content to let people live without their basic needs being met, rather than investing the necessary resources into social programs:

The government, the wacko conservatives, especially, really like to force people to have babies, bring more and more babies into the world, but then the help gets cut off when it’s a real person that needs things when it’s really alive. It makes me so mad. We’re like a fetus-loving culture but then we treat real children like shit…. And the government seems fine in just letting it happen. Letting people live in absolute shit and filth with nothing, not even basics (Cruz).

In addition to the inadequacy of supports for economically marginalized people, many participants highlighted systemic problems with accessing health care, describing the barriers YEH face in accessing health care, and the realities of class-based health inequities. For instance, one participant described the injustice of health-care inaccessibility: “I mean, everybody–any country that doesn't take care of its sick or doesn't let people have access to health care I think is committing a crime, so it’s ridiculous” (Hira). Several participants described the difficulties of navigating the complexities of the health care system. Finding health insurance was often described as “confusing,” with one participant speculating that “Like insurance or doctors or housing vouchers. All of that is almost on purpose confusing, maybe just so we don’t find out about them and so we can’t end up using any of those things available” (Cruz).

Some participants identified that YEH and economically marginalized youth have less access to information about reproductive health than their more privileged peers, referring to YEH as “forgotten” and “trapped.” For instance, one participant stated,

I think a lot of my future is really bright and hopeful but I admit that I just don’t know about so many things that are sort of obvious to people who have more money or who haven’t had life challenges that I have (Jadyn).

### Theme 3: YEH Expressions of Within-Group Stigma

#### Within-group stigma, othering, and role distancing

Within-group stigma, often referred to as “intragroup stigma,” is the process by which stigmatized individuals apply the pejorative labels assigned to them towards those who are more evidently stigmatized (Gunn & Canada, 2015). As discussed in the section about pregnancy stigma above, some participants engaged in within-group stigmatizing within the interviews, repeating dominant pejorative narratives and labels about other YEH. Participants often distanced themselves from these negative labels and stereotypes by describing a multiplicity of stories, experiences, and truths within themselves, while describing other YEH in one-dimensional stereotypical ways.

Typical labels that participants assigned to other YEH included laziness, immaturity, perceived invincibility, impulsivity, and poor judgment. In addition to pregnancy, these labels typically were assigned when discussing topics of family planning, and condom and contraception use. For instance, “Yeah, I think [other YEH] live in a bubble and assume that nothing bad could be going on with them because they’re too young, and pregnancy or diseases or life-threatening things just don’t really happen to young people” (Moana). Several participants described YEH as immature, with one participant stating:

I think they need to just know more, and why it matters for them to know and care about these things, because they’re not as invincible as they think. Like, your life is not just like a video game or something that you get to restart if you fall in a hole. So they just need more awareness and maturity. Most of the people in the shelter, I will tell you, are acting more like 12 year-olds… (Arya).

Many of the participants applied common homelessness stereotypes to other YEH, while positioning themselves as resisting these narratives. For example, in response to a question regarding what would help YEH, one youth described,

I think [YEH] need to find a way to improve their self-esteem and stay accountable to goals in a way that isn’t tied to pregnancy…and not just be some homeless person who is trying to game the system or tie down a relationship by getting pregnant…(Karsyn).

This participant positioned himself as an exception from YEH stereotyping and stigma, which did apply to other YEH. This perspective appears to endorse the dominant neoliberal narratives of hard work yielding positive results, while YEH not pursuing upward mobility through success are deemed to be deficient in intrinsic ability or willingness, and therefore deserving of the stigma assigned to them.

#### Counter-narratives of empathy

In contrast to instances of within-group stigma, some participants also expressed empathy towards their peers. For instance, while many participants were critical of YEH who viewed pregnancy as a means to access more resources, other participants described the same strategy as understandable within the context of the scarcity of supports for YEH:

And how could you blame anyone here for wanting those things? People are desperate, and homeless, and willing to do whatever just to be able to see doctors, get housing, get things for free, because they have nothing. It’s a horrible life. Homelessness is possibly worse than death (Cruz).

Similarly, while many participants criticized YEH as “lazy” for not using birth control, ascribing this to inherent individual failures, other participants expressed empathy and understanding for how systemic barriers may shape people’s access to sexual and reproductive health-care knowledge and resources. Another participant described,

…most people [YEH] haven’t had a lot of education about any of this. Like healthy sexual health things, and like pregnancy, and getting tested. Just sex things in general. Because a lot of us moved around a lot growing up. Some were in foster care, some with a lot of placements. And some didn’t finish school or nothing like that either. So if parents or like their foster parents didn’t really talk about it, then they maybe don’t know everything they should to be healthier about that (Ale).

Rather than portraying YEH as “lazy” and “dirty” and asserting that not being diligent about contraception and sexual health testing as an intrinsic moral failing on the part of the individual, this participant articulated empathy and understanding for why YEH make the choices that they do within a broader systemic context which does not provide opportunities for YEH to obtain the information and resources they need.

Many participants discussed the effects of their intersectional identities and articulated an awareness of how broader social, economic, and political forces contributed to the stigma faced by YEH, demonstrating a reluctance to subscribe to dominant narratives surrounding youth homelessness. One participant stated that politicians who stated that they “only like a certain type of skin color… [The United States is] for everyone- not just for a certain race… [racist politicians] are trying to repeat a time that was very detrimental to the universe. To everybody” (Hira). It appears that the lived experiences of these participants facilitated awareness of systemic barriers to upward mobility. The process of externalizing problem-saturated stories and connecting individual experiences with the broader social context provided a counter-narrative leading to further empathy and understanding.

#### Expressions of ambivalence in youth experiencing homelessness narratives

Despite the polarity in views described, most participants vacillated between feeling empathy towards other YEH and expressing internalized judgment when sharing their own narratives and explaining reproductive and sexual health care among YEH. Within single interviews, multiple positive and negative narratives about YEH were often expressed. These juxtaposing narratives were seen especially in relation to reproductive health. One participant described other YEHs’ attitudes towards receiving regular gynecology check-ups: “And some just think nothing will ever happen specifically to them…the mentality is that people would rather get drunk or high every single night and hope for the best, that the pregnancy ends. Or that the pregnancy won’t somehow be affected…” (Jae). Simultaneously, this participant expressed empathy for fellow YEH by expressing an understanding for the unique stress that homelessness brings: “And because I think a lot of people are like, ‘I just don’t have the emotional stamina to figure this out today.’ That’s how I feel a lot, actually” (Jae).

## Discussion

Findings of this study highlight the importance of allowing YEH to share and shape their own narratives around experiences of stigma and resilience. Throughout the interviews, participants repeatedly identified stigma and prejudice that they experienced socially, both within and outside of the homelessness contexts. The results of this study mirror other research demonstrating that YEH experience stigmatizing attitudes that result in stress and less access to resources. As well, this study contributes a more nuanced understanding of how YEH actively resist stigma and challenge dominant societal perceptions within their everyday lives, and with regard to narratives pertaining to reproductive health, pregnancy, and parenting. Several important considerations have been garnered through these research efforts.

### Onus of Homelessness Responsibility from the Self to the System

Participants considered their experiences of stigma within the larger systemic forces that create homelessness and castigate YEH. The research team interpreted the rich descriptions provided by participants as insights into the societal forces that have resulted in conditions of oppression for YEH. It was discussed whether participants’ identification of systemic oppression should be considered resistance to stigma, as for YEH, such oppression shapes daily life and may seem obvious. We therefore reflected on whether our interpretation was a result of our own privileges and social work research lenses. However, the team ultimately agreed that participants’ abilities to contextualize their own hardships within broader systems of oppression may provide powerful counter-narratives that defy stigmatizing narratives about homelessness being a result of individual failures. YEHs’ abilities to shift narratives of “brokenness” from themselves to the system allows youth to see the complexity of the world and serves to decrease self-blame; as extant research suggests, such self-blame is associated with negative mental health outcomes (Kidd, 2007). These findings thus provide further evidence that supporting YEH in developing their understanding of the impacts of systemic discrimination may play a protective role against the internalization of stigma and have beneficial impacts on mental health (Kidd, 2007). This study builds on prior research examining YEHs’ structural counter-narratives on homelessness (Toolis & Hammack, 2015), also contributing new understanding of how stigma related to reproductive and sexual health may intertwine with other stigmas among YEH, eliciting processes of both internalization and resistance that impact identity formation.

### Stigma and Identity Formation

Identity formation is an important task of adolescence and young adulthood (Erikson, 1968). The factors associated with homelessness, poverty, and reproductive health can complicate these processes during this sensitive developmental time. As with all youth, participants discussed processes involved with developing a concise sense of self and identity; however, they also discussed additional tasks of learning how to mediate the stigma of having homelessness be an aspect of one’s identity. This struggle may be experienced more acutely by YEH with intersectional identities, such as LGBTQ+ YEH, whose social environments may pose additional barriers to identity formation and expression (Schmitz & Tyler, 2018). This process can shape a youth’s ability to adjust to and see themselves as valuable members of their social environment. Youth homelessness is associated with feelings of being an outsider and social disequilibrium (Auerswald & Eyre, 2002). The creation of buffers against stigma and negotiations of which narratives YEH choose to internalize appears to be a way that participants have learned to cope with the developmental impact of youth homelessness. As homeless identities are generally constructed by individuals who are not homeless, YEH are at risk of becoming *objects of discourse* (McCarthy, 2013) rather than self-defined. This research echoes other accounts of YEH engaging in self-definition and articulation of more accurate narratives (Jackson, 2015; Kidd et al., 2017). As well, the role of pregnancy and sexual health in the lives of participating YEH resulted in the creation of a new source of strength and pride in relation to their identities: that of competent parents and/or sexually self-efficacy. These results parallel the work of Edin and Kefalas (2011), whose documentation of the experiences of poor single mothers in urban centers of the USA found that single motherhood became a source of pride for their participants, and their ability to parent their children well despite various economic and societal barriers. Also congruent with Edin and Kefalas’ (2011) narratives of poor single mothers, YEH described the redemptive and rewarding experiences that parenting while homeless has offered them, contrary to the more dominant stigmatizing narratives about youth homelessness and pregnancy. This research contributes to growing literature on homelessness and self-definition (Farrugia, 2011; Parsell, 2011), while also adding the complicating factor of youth pregnancy and parenting, another identity formation and development that YEH are interpreting through counter-narratives.

### Youth Experiencing Homelessness Expressions of Empathy and Systemic Context

Study findings also demonstrate that many participants were able to see themselves beyond stigma and resituate the cause of homelessness within systems of oppression. Within this process, many participants expressed empathy and caring for others going through similar experiences to them. A significant area of research has been concerned with demonstrating the importance of empathy expressed by service workers (Collins & Barker, 2009; Hudson, et. al, 2007; Thompson, et. al, 2006). Therefore, documenting expressions of in-group empathy is an important contribution to the YEH literature, as YEH have expressed the importance of social awareness and out-group empathy towards YEH (Barman-Adhikari et.al, 2019). Social empathy has generally been applied as a framework to youth programming (Wagaman, 2011), demonstrating that YEH programming can draw on the empathy already being felt and expressed by YEH towards others as a method for empowerment.

### Internalized and Within-Group Stigma

Despite the finding that participants empathized with and understood the struggles of other YEH, there were examples of internalized deficit-based narratives of homelessness in line with neoliberal conceptualizations of poverty. The neoliberal welfare state is comprised of political economic practices that emphasize the liberation of individual entrepreneurial freedoms such as free markets and trade (Harvey, 2005). Neoliberalism emphasizes the importance of placing responsibility for social processes, such as income, property ownership, and economic scarcity on individual behaviors (Hennigan, 2017). This applies to homelessness, in which the responsibility for both becoming poor and staying homeless is placed on the individual, absolving the state of any social responsibility. The findings of this study demonstrated an application of internalized neoliberal conceptualization of responsibility, which were applied by some YEH to other members of the homeless community, especially in regard to pregnancy and sexual health decisions. This perspective conceptualizes other YEH as either resistant to, or unable to, engage in productive behaviors (Weng & Clark, 2018).

The finding that participants expressed dominant narratives of stigma and discrimination may serve as a defense mechanism whereby YEH distance themselves from others. It may also be interpreted as an egoistic developmental process indicative of adolescence in which youths’ world-views are more self-focused. The purpose of this distancing requires further research, while offering a powerful example of the insidiousness of dominant narratives and a demonstration of diversity within the homeless youth population. YEH interactions with stereotypes and stigma, which include rejection and enactment, have been documented (Harter, et. al, 2005). However, future work can further elucidate the purpose of within-group stigma for YEH. Goffman (1975) concept of role distance is marked by the separation between an individual and their role through behaviors that denote a dissatisfaction and resistance against the role. YEHs’ role distancing may serve a protective function by guarding their own self-image and distinguishing themselves as exceptions to stigmatizing stereotypes.

### Research Limitations

The results of this study may be limited as the YEH who participated in this research were comprised of service-seeking youth only, preventing transferability to youth without access to or who are reticent to engage with services. As well, the research was completed in a dense, urban environment, producing results specific to this setting. Rural and smaller communities that do not have as many services in comparison to the study’s urban and more service-rich context require further research in this area. Although the participants in this study are relatively diverse, findings related to various intersectional identities of YEH (i.e., ethno-racial identities and LGBTQ+ status) should be further explored. Another limitation of this research is related to the subsequent analyses of the qualitative interview data, which were completed after the original interviews were collected and analyzed. As the interviews and original analysis were completed with another purpose in mind, the secondary finding of YEH resilience and counter-narrative should be further investigated and replicated as the focal point of future research.

### Implications for Practice

This study provides a YEH-centered and strengths-based perspective of resilience and resistance to stigmatizing narratives related to homelessness and reproductive and sexual health. For individuals who work with YEH, these findings may assist workers in becoming more attuned to positive narratives of resilience and the many aspects of identities that YEH embody beyond the presenting clinical concerns. As YEH are identifying socio-political forces that require change, practitioners need to become more attuned to engaging clients in discussions of politics and finding ways to assist their clients to engage in resistance against broader systemic forces that promulgate youth homelessness. For clinicians working with YEH in a therapeutic context, modalities that focus on establishing YEHs’ stories about themselves and experiences may be particularly salient, such as narrative therapy and arts-based activities. Although this approach has been described in work with youth in a variety of treatment settings, further intervention research is required to understand the effects of narrative approaches and their applicability to YEH and sexual health. Narrative approaches offer YEH a means to combat stigma and discrimination, elevate the preferred stories of youth, and see youth as more than the stigmatizing narrative assigned to them.

As well, professionals can engage in advocacy, research, and policy work to address the systemic context that plays a role in the development and maintenance of stigma, and the anti-homeless policies stemming from it. Counter-narratives are important for YEH, workers, and policy makers to better understand experiences of youth homelessness and intersections with pregnancy and sexual health. Though it is of great importance to amplify the voices and resistance of YEH in all dimensions of research, service provision transformation, and policy change, the onus for dismantling structural inequity does not lie solely within YEH; social workers, frontline service-workers, advocates, community members, and policy decision-makers alike must step up as part of the process of influencing change and disrupting the root causes of these oppressions and stigmas that so disproportionately harm young people. As such, we join with Aparicio et al. (2021), in calling for structural interventions to address the financial instability and lack of affordable housing which prevent youth from maintaining housing, beginning with a universal basic income program, and a Housing First approach which prioritizes stable housing for all as the first necessary step toward ending homelessness.

Finally, the dominance of participant narratives about experiences of stigma within health-care settings in this study demonstrate the role which health care providers can play in contributing to the harm of social stigma, thereby creating barriers which prevent YEH from accessing the reproductive, sexual, and other health information and supports that they need. These narratives serve as a powerful reminder to all health and service providers of the importance of meeting every client with respect and empathy and providing non-judgmental care.
